# Study of Duguetia furfuracea aqueous extract treatment in metabolic profile and renal fuction biomarkers of obese Wistar dams

**DOI:** 10.1186/1758-5996-7-S1-A125

**Published:** 2015-11-11

**Authors:** Dener Lucas Araújo dos Santos, Dionys de Souza Almeida, Aline Zanatta Schavinski, Amanda Lima Deluque, Eduardo Kloppel, Carolina Abreu Miranda, Kleber Eduardo de Campos

**Affiliations:** 1 Universidade Federal de Mato Grosso, Barra do Garças, Brazil

## Background

Obesity is a pathophysiology condition that influences the metabolic pathways, and the association with pregnancy can worsen the lipidic and glycemic profiles. Moreover, Duguetia furfuracea has been used for obesity and also kidney disease treatment, however a very few studies have been conducted to verify its effectiveness and safety for using as well as to evaluate its influence on metabolic profile.

## Objective

To evaluate the biomarkers for metabolic profile and renal function of aqueous extract treatment of D. furfuracea in obese Wistar dam rats.

## Materials and methods

Ten newborn female Wistar rats were used, were administrated monosodium glutamate solution, 4.0 mg/Kg body weight (obese) in neonatal period. At adult age (90 days of life) these female rats were mated with healthy male rats and after offspring weaning period, the dams were used and divided into two groups: control (CONT; n=05) daily treated with water; and experimental (DF n=05) daily treated with aqueous extract of D. furfuracea at a dose of 200 mg/Kg, both treatment for 28 days. Lee Index for obesity classification was measured in first and last day of experimental protocol, and weekly it was measured body weight, blood glucose, urine flow, fecal weight, and food and water consumption. At 28th day of treatment, all rats were anesthetized and killed by decapitation and thus serum biomarkers were measured [total protein, cholesterol and its fractions (HDL, LDL and VLDL), triglycerides and liver transaminases (ALT and AST). Moreover, several organs were collected (liver, kidney and periovarian fat) and also performed their relative weight. Statistical significance was p <0.05.

## Results

Obesity was confirmed by Lee index, showing higher values of 0.300, demonstrating the success of obesity induction method. Food intake was increased in days of treatment 7, 14, 21 e 28 in CONT dams, compared to first day (zero). When both groups were compared, the DF dam rats presented a higher food intake compared to CONT dams at day 0. The other parameters did not presented changes during all experimental period, nor the biochemical evaluations.

## Conclusion

The dose used in treatment of D. furfuracea in experimental obesity did not change the metabolic profile, nor presented alteration in renal function. Moreover, it did not any present toxic effect.

